# Integrating Gaucher disease (GD) into mainstream hematological curriculum training

**DOI:** 10.1002/hem3.70474

**Published:** 2026-10-01

**Authors:** Colm Bradley, Argiris Symeonidis, Sam Salek, Nkemdirim Jacob

**Affiliations:** ^1^ University of Glasgow Glasgow United Kingdom; ^2^ University of Patras Patras Greece; ^3^ University of Hertfordshire Hertford United Kingdom; ^4^ Queen Elizabeth University Hospital Glasgow United Kingdom

Gaucher disease (GD) is the most common lysosomal storage disorder and arises from pathogenic variants in *GBA1* that reduce glucocerebrosidase activity, leading to lysosomal accumulation of glucosylceramide and related metabolites in macrophages.^1^ Although rare, GD has major relevance to hematology because many of its earliest and most persistent manifestations fall within routine hematology practice, including splenomegaly, anemia, thrombocytopenia, bleeding symptoms, marrow infiltration, paraproteinemia, and skeletal complications that may mimic malignant or inflammatory disease.^2^ Delayed diagnosis remains common, and surveys suggest that awareness among hematologists is better than in some other specialties but still incomplete, especially when disease presents atypically or with mild phenotypes.^3^ This creates a strong case for formal integration of GD into mainstream hematology training.

The purpose of this editorial is to define the core knowledge and clinical competencies that hematology trainees should acquire. The emphasis is not that every hematologist must become a GD subspecialist, but that all should be able to recognize when to suspect the disease, request appropriate first‐line investigations, interpret key laboratory and imaging findings, understand modern treatment principles, and work effectively with specialist centers. This is particularly important in Europe, where training standards are increasingly harmonized and where rare disease diagnosis depends on shared awareness rather than isolated expertise.

## CORE SCIENTIFIC KNOWLEDGE

Trainees should understand the basic biochemistry and cell biology of membrane sphingolipids, lysosomal degradation pathways, and the role of glucocerebrosidase and its cofactors, including saposin C.^4^ They should know that hundreds of pathogenic *GBA1* variants exist, that genotype influences phenotype, but does not fully determine it, and that common variants, such as N370S and L444P differ in their typical clinical associations.^5^ Knowledge of lysosomal dysfunction is clinically relevant because substrate accumulation and secondary metabolites, such as glucosylsphingosine contribute to chronic inflammation, immune dysregulation, osteoclast activation, coagulation abnormalities, and neurodegeneration. Macrophage activation is central to disease pathophysiology, affecting the liver, spleen, bone marrow, and, in some forms, the nervous system and lungs.

## EPIDEMIOLOGY AND DISEASE SPECTRUM

Hematologists should know that GD is pan‐ethnic but enriched in Ashkenazi Jewish populations and is substantially underdiagnosed worldwide, particularly in milder type 1 disease.^6^ Classical clinical classification distinguishes type 1 (nonneuronopathic), type 2 (acute neuronopathic), and type 3 (chronic neuronopathic) disease, but clinicians should also recognize perinatal lethal presentations, hydrops fetalis, and rare syndromic phenotypes involving cardiac, pulmonary, or cutaneous disease.^7^ Because phenotype varies widely, education should focus on recognizing patterns rather than relying on a single textbook description.

## CLINICAL MANIFESTATIONS RELEVANT TO HEMATOLOGY

From a hematology perspective, the most important presenting features are splenomegaly, hepatomegaly, thrombocytopenia, normocytic anemia, bleeding symptoms, hyperferritinemia, inflammatory markers, bone pain, osteopenia, avascular necrosis, and sometimes monoclonal gammopathy.^1^ Patients may also report fatigue, growth delay, abdominal discomfort, easy bruising, epistaxis, or early satiety. Bone disease may range from asymptomatic marrow infiltration to recurrent bone crises and fractures, and pulmonary or neurological manifestations may be clues in multisystem disease.^8^ Hematologists should remember that GD can resemble more common disorders such as immune thrombocytopenia, hypersplenism of other causes, hemoglobinopathies, chronic liver disease, leukemia, lymphoma, myeloma, or metastatic bone disease.^1^, ^3^

## LABORATORY AND DIAGNOSTIC APPROACH

The curriculum should teach a practical diagnostic pathway. Initial assessment should include full blood count, blood film, inflammatory and biochemical markers, coagulation studies where indicated, and imaging of liver, spleen, and skeleton according to presentation. Biomarkers such as chitotriosidase, CCL18/PARC, and especially glucosylsphingosine (Lyso‐Gb1) are useful for supporting diagnosis and monitoring disease burden. Definitive diagnosis relies on demonstration of reduced glucocerebrosidase activity, followed by molecular confirmation of the underlying pathogenic variants. Bone marrow examination is usually unnecessary for diagnosis and should not be used routinely, when non‐invasive testing is available; if Gaucher cells are seen incidentally, they should prompt confirmatory enzyme and genetic testing rather than substitute for it. Training should also cover baseline assessment of disease burden and complications. This includes organ volume measurement, MRI or other appropriate imaging for marrow and bone involvement, bone mineral density assessment, and screening for coagulopathy, monoclonal gammopathy, pulmonary disease, and other organ‐specific complications where clinically relevant.^8^, ^9^ Trainees should understand that no single biomarker captures the full disease phenotype; interpretation must integrate clinical findings, laboratory markers, imaging, and longitudinal change.

## DIFFERENTIAL DIAGNOSIS AND CASE‐FINDING

Educational programs should emphasize the situations in which GD should enter the differential diagnosis: unexplained splenomegaly, cytopenias, bleeding disproportionate to platelet count, childhood growth failure, unexplained bone lesions, marrow histiocytes, small paraproteins with splenomegaly, or early‐onset Parkinsonian features in the right clinical context.^1^, ^2^ Because missed diagnosis often reflects failure to consider the disease at all, simple diagnostic algorithms and point‐scoring tools may help non‐specialists identify patients who need confirmatory testing.^10^

## TREATMENT PRINCIPLES

Hematology trainees do not need to master every therapeutic protocol, but they should understand the goals, indications, and limitations of current therapy. Treatment aims to reverse or stabilize organomegaly, cytopenias, skeletal disease, growth impairment, fatigue, and other complications while preventing irreversible damage.^11^ The two main disease‐specific approaches are enzyme replacement therapy (ERT) and substrate reduction therapy (SRT). ERT has transformed outcomes in type 1 GD and remains a mainstay of care, while oral SRT offers an alternative for selected patients.^12^, ^13^ Emerging strategies, including pharmacological chaperones, are also relevant because they illustrate how the therapeutic landscape is evolving.^14^ Trainees should know that treatment choice depends on phenotype, severity, comorbidity, patient preference, access, and specialist guidance. Supportive management is an equally important educational theme. This includes treatment of anemia and bleeding, orthopedic management of fractures or osteonecrosis, bone health optimization, management of infection risk, and psychological support for patients living with a chronic inherited disorder. Hematologists should also understand when splenectomy is inappropriate or should be avoided, and when coordination with orthopedics, neurology, hepatology, pulmonology, genetics, pediatrics, and specialist nursing is essential.

## MONITORING AND LONG‐TERM FOLLOW‐UP

Long‐term care requires structured monitoring. Trainees should learn how biomarkers, such as chitotriosidase, CCL18/PARC, and Lyso‐Gb1 can be used alongside blood counts, organ imaging, skeletal evaluation, and assessment of patient‐reported symptoms to judge response or progression. Follow‐up should be individualized, but it must remain vigilant for complications such as persistent thrombocytopenia, progressive bone disease, pulmonary hypertension, neurological deterioration in neuronopathic forms, and treatment‐related issues. Importantly, apparent hematologic improvement does not exclude ongoing skeletal or systemic disease, so monitoring must remain multidisciplinary.

## LINKS WITH OTHER DISEASES AND GENETIC COUNSELING

A modern curriculum must also address the wider clinical significance of GD and *GBA1*. Trainees should know the established association between *GBA1*‐related biology and Parkinson's disease, as well as broader links with neurodegeneration and inflammation. For hematologists, the association with monoclonal gammopathy and multiple myeloma is particularly important, because chronic immune stimulation and bioactive lysolipids may contribute to clonal plasma cell disorders. Increased risk of other hematologic malignancies and some solid tumors has also been reported, which reinforces the need for ongoing surveillance and careful interpretation of paraproteins, marrow findings, and unexplained constitutional symptoms.^15^ Genetic counseling is another essential competency. Hematologists involved in diagnosis should understand autosomal recessive inheritance, carrier detection, family screening, and the principles of prenatal and preconception counseling. Even when formal counseling is delivered by genetics services, hematologists are often the first clinicians to explain why family members may need testing and how diagnosis affects reproductive planning.

## IMPLICATIONS FOR TRAINING

The educational objective is therefore, practical and competency based. A trainee in hematology should finish training, able to suspect GD in relevant clinical scenarios, request and interpret first‐line tests, understand classification and major complications, communicate effectively with patients and families, and liaise appropriately with expert centers. Programs should combine didactic teaching with case‐based learning, exposure to diagnostic algorithms, and, where possible, short placements in specialist GD services for trainees, based in centers without local expertise. This would align with broader European efforts to harmonize hematology education and to improve care for rare but treatable disorders.

## SUMMARY AND CONCLUSIONS

GD should be embedded within mainstream hematology training because its core manifestations frequently present to hematologists, its diagnosis is often delayed, and effective therapies are available. The minimum curriculum should cover disease biology, epidemiology, phenotypic diversity, hematologic and systemic manifestations, practical diagnostic testing, treatment principles, monitoring, links with malignancy and neurodegeneration, and family counseling. Integrating this content into hematology training would improve case recognition, reduce avoidable diagnostic delay, strengthen multidisciplinary care, and support more equitable access to specialist management. For centers without direct experience, structured links with expert GD services should be part of training delivery. In this way, hematology education can better reflect the realities of rare disease practice and ensure that trainees are prepared to identify and manage Gaucher disease within routine clinical care.

## AUTHOR CONTRIBUTIONS

**Colm Bradley:** Writing—review and editing; conceptualization; writing—original draft. **Argiris Symeonidis:** Writing—review and editing; conceptualization. **Sam Salek:** Writing—review and editing; conceptualization. **Nkemdirim Jacob:** Writing—review and editing.

## CONFLICT OF INTEREST STATEMENT

The authors declare no conflicts of interest.

## FUNDING

This research received no funding.
